# PPAR-α acutely inhibits functional activity of ASICs in rat dorsal root ganglion neurons

**DOI:** 10.18632/oncotarget.21805

**Published:** 2017-10-10

**Authors:** Jing Wu, Jia-Jia Wang, Ting-Ting Liu, Yi-Mei Zhou, Chun-Yu Qiu, Ding-Wen Shen, Wang-Ping Hu

**Affiliations:** ^1^ Research Center of Basic Medical Sciences, School of Basic Medical Sciences, Hubei University of Science and Technology, Xianning 437100, Hubei, P.R. China; ^2^ Department of Physiology, School of Basic Medical Sciences, Hubei University of Science and Technology, Xianning 437100, Hubei, P.R. China; ^3^ Department of Pharmacology, Hubei University of Science and Technology, Xianning 437100, Hubei, P.R. China

**Keywords:** peroxisome proliferator-activated receptor-α, acid-sensing ion channels, proton-gated current, nociception, dorsal root ganglion neuron

## Abstract

Peroxisome proliferator-activated receptor-α (PPAR-α), a lipid activated transcription factor of nuclear hormone receptor superfamily, can relieve pain through a rapid-response mechanism. However, little is known about the underlying mechanism. Herein, we report that PPAR-α activation acutely inhibits the functional activity of acid-sensing ion channels (ASICs), key sensors for extracellular protons, in rat dorsal root ganglion (DRG) neurons. Pre-application of PPAR-α agonist GW7647 for 2 min decreased the amplitude of proton-gated currents mediated by ASICs in a concentration-dependent manner. GW7647 shifted the concentration-response curve for proton downwards, with a decrease of 36.9 ± 2.3% in the maximal current response to proton. GW7647 inhibition of proton-gated currents can be blocked by GW6471, a selective PPAR-α antagonist. Moreover, PPAR-α activation decreased the number of acidosis-evoked action potentials in rat DRG neurons. Finally, peripheral administration of GW7647 dose-dependently relieved nociceptive responses to injection of acetic acid in rats. These results indicated that activation of peripheral PPAR-α acutely inhibited functional activity of ASICs in a non-genomic manner, which revealed a novel mechanism underlying rapid analgesia through peripheral PPAR-α.

## INTRODUCTION

Peroxisome proliferator-activated receptor-α (PPAR-α), a lipid activated transcription factor, belongs to a nuclear hormone receptor super-family. It is activated by endogenous ligands, such as palmitoylethanolamide (PEA) and oleoylethanolamide (OEA) [1]. And it can be activated by some synthetic drugs, such as GW7647 and Wy-14643. PPAR-α is involves in peripheral pain since its expression in dorsal root ganglia (DRG) neurons [1, 2]. Clinical evidence shows that the endogenous PPAR-α agonist, PEA, is effective in alleviating various human pain, even in patients who failed to other treatment [3–6]. Animal research has also provided analgesic properties of both synthetic and natural ligands to PPAR-α in several models of inflammatory, visceral, and neuropathic pain. For example, administration of PPAR-α agonists (e.g., GW7647, PEA, Wy-14643 and OEA) can suppress inflammatory pain elicited by carrageenan, formalin and complete Freund's adjuvant, reduce visceral nociception induced by magnesium sulfate and acetic acid, prevent mechanical and thermal hyperalgesia evoked by nerve injury [2, 7, 8]. These effects are absent in PPAR-α-null mice and prevented by the PPAR-α antagonist in rats. PPAR-α-null animals are insensitive to the antinociceptive effects of PPAR-α agonists to various proalgesic and proinflammatory stimuli [9]. In addition, elevation of local endogenous PEA can also attenuate inflammatory and neuropathic pain through PPAR-α [10, 11]. As a transcription factor, PPAR-α activation can repress expression of genes for inflammatory mediators involved in pain, such as proinflammatory cytokines, by a molecular mechanism termed ligand-dependent direct transrepression [12]. Because the transrepression unfolds over a period of hours or even days, it cannot account for the fast analgesic effects of PPAR-α agonists on nocifensive behaviors, which occur within minutes of drug administration. PPAR-α agonists can relieve pain rapidly but transiently (minutes-hours), indicating that they may act at non-transcriptional targets through a rapid-response mechanism [2, 13]. However, the exact non-genomic mechanisms by which PPAR-α agonists relieve pain remain unclear.

Acid-sensing ion channels (ASICs) are a family of proton-sensing channels. they are activated by lowering extracellular pH. So far, at least six different ASIC subunits derived from 4 genes have been found in mammals [14]. Most of the ASIC subunits (i.e. ASIC1a and b, ASIC2a and b, and ASIC3) are expressed in both DRG cell bodies and peripheral terminals, which contribute to proton-induced pain signaling [15–18]. It is known that pain can be produced by tissue acidosis. Protons depolarize DRG neurons and generate action potenials through activating ASICs. For instance, direct application of an acidic solution into the skin induces non-adapting pain [19]. A recent report shows that inhibition of TRPV1, TRPA1 or ASICs does not alter pain induced by intraepidermal injection of pH 4.3 in human [20]. However, most studies demonstrate that peripheral moderate pH (up to pH 6.0) produces pain sensation through activating ASICs, rather than TRPV1 [21–23]. ASICs have been believed to be involved in pain sensation under conditions of tissue acidification, such as lesions, inflammation, ischemia, and tumors. Among the ASIC subunits, ASIC3 subunit is found to localize in nociceptive fibers [24, 25]. ASIC3 is also one of the most sensitive ASIC subunits, which can sense a decrease of pH to around pH 7.2 [23, 26]. Activating ASIC3 has been found to contribute to the generation of pain in primary sensory neurons. Blocking ASIC3 at the periphery inhibits the spontaneous pain generated by mild cutaneous acidification, reverses CFA-induced primary hyperalgesia and reduces post-operative pain behaviors when applied to the incised area during surgery [16, 27, 28]. There is increasing evidence that ASICs play a significant role in many types of pain, such as inflammatory and postoperative pain [17, 28]. ASICs have thus become important therapeutic targets for treating pain.

Herein, we report that PPAR-α activation acutely inhibits the activity of ASICs in nociceptive DRG neurons and relieves acidosis-induced pain in rats.

## RESULTS

### Inhibition of the proton-gated current by PPAR-α agonist in rat DRG neurons

**Figure 1:**
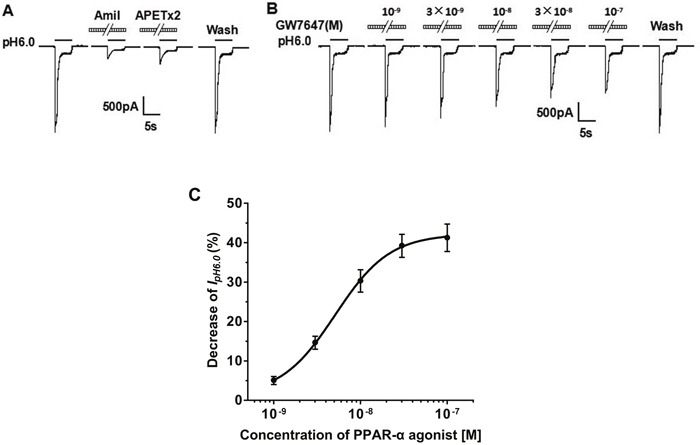
Concentration-dependent inhibition of the proton-gated currents by PPAR-α agonist in rat DRG neurons The proton-gated currents were recorded in the presence of capsazepine (10 μM) or AMG 9810 (1 μM) to block proton-induced TRPV1 activation. **(A)**. Representative traces show currents evoked by a pH 6.0 acidic solution for 5 s in rat DRG neurons with membrane potential clamped at-60 mV. The proton-gated currents could be completely blocked by 100 μM amiloride (Amil), a broad-spectrum ASIC channel blocker. It was also blocked by 3 μM APETx2, an ASIC3 blocker. **(B)**. Sequential current traces illustrate the inhibition of proton-induced currents by different concentration of GW7647 (10^−9^ M - 10^−7^ M) and recovery after washout of GW7647, a PPAR-α agonist. Representative currents were recorded for more than 60 min in a DRG neuron tested. GW7647 was pre-applied to external solution for 2 min. **(C)**. The graph shows GW7647 decreased the peak amplitude of proton-gated currents in a concentration-dependent manner with an IC_50_ of 5.11×10^−9^ M. Each point represents the mean ± SEM of 8-10 neurons.

Isolated rat DRG neurons in small and medium diameter (15 to 35 μm) were used to current measurements in the present study. To purify the ASIC currents from the proton-activated currents, we blocked proton-induced TRPV1 activation by adding capsazepine (10 μM) or AMG 9810 (1 μM) to external solution [29, 30]. As described, lowering extracellular pH from 7.4 to 6.0 for 5 s caused a rapid inward current (I_pH6.0_) in most DRG neurons tested (74.7%, 112/150) [31, 32]. There is no difference in magnitude or shape of I_pH6.0_ in the presence of capsazepine (10 μM) or AMG 9810 (1 μM). The I_pH6.0_ could be completely blocked by amiloride (100 μM), a broad-spectrum ASIC channel blocker (Figure 1A). Thus, The I_pH6.0_ was considered to be ASIC currents. Most (80.4%, 90/112) of these ASIC currents were characterized by a large transient component followed by fast inactivation and then a small sustained component with no or very slow inactivation [33]. As shown in Figure 1A, these ASIC currents were completely blocked by APETx2 (3 μM), a specific ASIC3 homomeric and heteromeric channel blocker. Thus, these proton-gated currents were mediated by ASIC3-containing channels. We observed the ASIC3-like currents only in the following study.

In the majority of the DRG cells sensitive to acidic stimuli (77.8%, 70/90), we found that pre-application of PPAR-α agonist GW7647, a synthetic PPAR-α agonist, for 2 min inhibited the proton-activated transient component of ASIC currents and did not alter sustained component (Figure 1). We first investigated whether the inhibition of proton-activated currents was dependent upon concentrations of GW7647. Figure 1B shows that the amplitude of peak I_pH6.0_ reduced with increase of concentration of pre-applied GW7647 (from 10^−9^ M to 10^−7^ M) in a representative DRG cell. After washout of GW7647, the inhibiting effect of GW7647 disappeared, suggesting a reversible effect. Figure 1C shows the concentration–response curve for GW7647 in the suppression of proton-activated currents. Maximum inhibiting effect (55.7 ± 8.2%, *n* = 8) of GW7647 occurred at concentration of 10^−7^ M. The half maximal inhibitory concentration (IC_50_) was 5.11 ± 0.16 nM (Figure 1C). The results suggested that GW7647 concentration-dependently inhibited ASIC currents.

### Concentration-response relationship for proton-gated currents with and without pretreatment of GW7647

**Figure 2:**
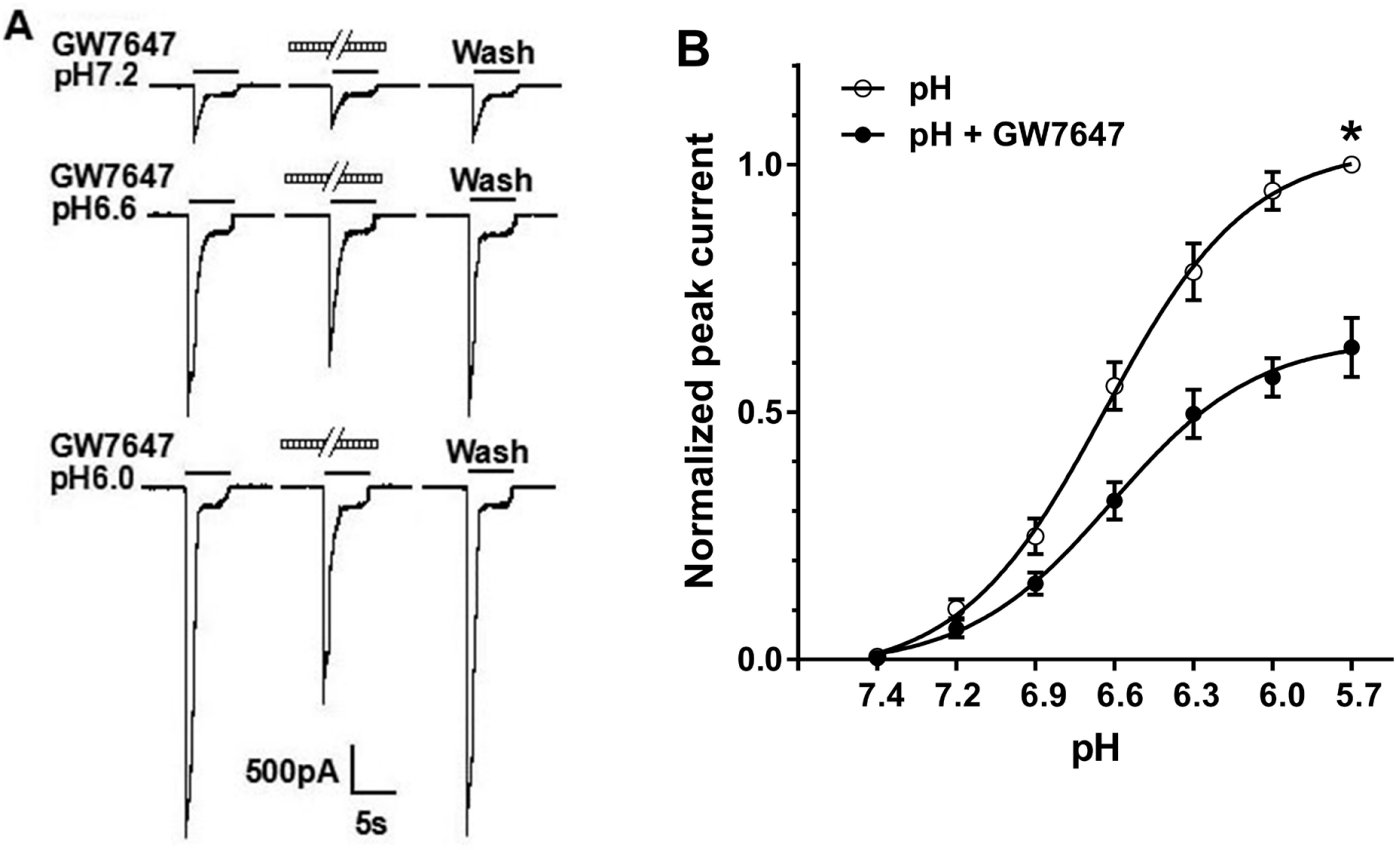
Concentration-response relationship for proton with or without the pre-application of GW7647 **(A)**. Sequential currents evoked by different pHs in the absence and presence of 3×10^−8^ M GW7647. **(B)**. The concentration-response curves for proton with or without 3×10^−8^M GW7647 pre-application for 2 min. The concentration-response curve for proton with morphine pretreatment shifted downwards. Each point represents the mean ± SEM of 8-10 neurons. All current values were normalized to the current response induced by pH 5.7 applied alone (marked with asterisk).

We then observed whether the suppression of GW7647 was dependent upon pH values. Figure 2A shows that pretreatment of GW7647 (3×10^−8^M) for 2 min decreased peak currents induced by three different pH values. Figure 2B shows the effect of GW7647 (3×10^−8^M) on concentration-response curve to protons. First, GW7647 shifted concentration-response curve to protons downwards. Maximal current response to protons decreased 36.9 ± 2.3% when GW7647 was pre-applied (*P* < 0.01, Bonferroni's post hoc test; Figure 2B). In contrast, the Hill coefficients or slopes of those two curves had no statistical difference (*n* = 1.54 ± 0.12 in the absence of GW7647 versus *n* = 1.55 ± 0.22 in the presence of GW7647; *P* > 0.1, Bonferroni's post hoc test). Second, the pH for half-maximal activation (pH_0.5_) values of both curves were not significantly different (pH_0.5_ of 6.65 ± 0.02 without GW7647 pretreatment versus pH_0.5_ of 6.62 ± 0.03 with GW7647 pretreatment; *P* > 0.1, Bonferroni's post hoc test). Finally, the threshold pH values of both curves were essentially similar in the presence and absence of GW7647.

### Involvement of PPAR-α in GW7647 inhibition of ASIC currents

**Figure 3:**
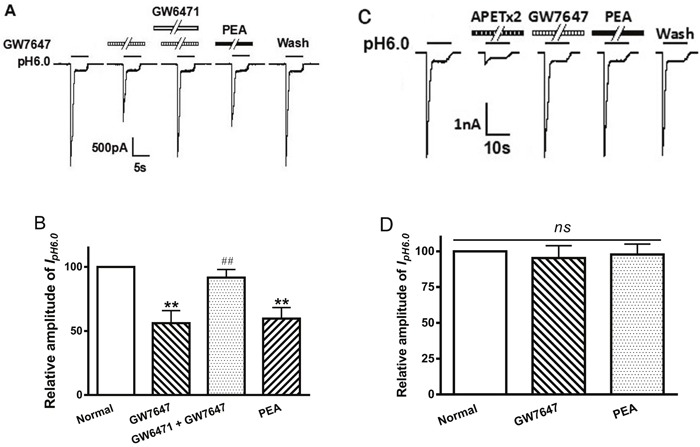
Involvement of PPAR-α in GW7647 inhibition of proton-gated currents The current traces in **(A)** and bar graphs in **(B)** show that the inhibiting effect of I_pH6.0_ by GW7647 (3×10^−8^M) pre-applied alone for 2 min in rat DRG neurons was abolished by the co-application of GW7647 and GW6471 (10^−7^ M), a selective PPAR-α antagonist, and mimicked by PEA (10^−5^ M), an endogenous ligand of the PPAR-α. Statistical tests were performed using one way analysis of variance followed by post hoc Bonferroni's test, and significance is shown as follows: ^**^P < 0.01, compared with normal column; ^##^*P* < 0.01, compared with GW7647 column. *n* = 10 in each column. The current traces in **(C)** and bar graphs in **(D)** show that GW7647 (3×10^−8^M) and PEA (10^−5^ M) had no effect on I_pH6.0_ in CHO cells expressing ASIC3 channel but not PPAR-α. Representative traces show currents evoked by a pH 6.0 acidic solution for 5 s in CHO cells expressing ASIC3. The proton-gated current could be blocked by 2 μM APETx2, a specific ASIC3 inhibitor. Currents were normalized to control (100%, white column). *n* = 10 in each column.

To verify whether the suppression of ASIC currents by GW7647 was mediated by PPAR-α, we examined the effect of GW6471, a selective PPAR-α antagonist, on the inhibition of GW7647. Figure 3A and B show that the inhibiting effect of GW6471 on I_pH6.0_ was reversed by the addition of GW7647. The peak amplitude of I_pH6.0_ decreased 43.87 ± 9.80% after pretreatment with GW7647 (3×10^−8^ M) alone for 2 min in ten DRG neurons (Figure 3). In contrast, GW7647 produced only a decrease of 9.33 ± 6.32% on I_pH6.0_ in ten neurons after the addition of GW6471 (10^−7^ M) (*P* < 0.01, Bonferroni's post hoc test; Figure 3). As an endogenous ligand of the PPAR-α, PEA was also pre-applied to DRG neurons tested. Similarly to GW7647, pretreatment of 10^−5^ M PEA for 2 min also caused a decrease of 40.34 ± 8.58% on I_pH6.0_ in ten DRG neurons (*P* < 0.01, Bonferroni's post hoc test; Figure 3). The results indicated involvement of PPAR-α in the suppression of ASIC currents in rat DRG neurons.

We also observed the effects of GW7647 and PEA on ASIC currents in CHO cells expressing ASIC3 but not PPAR-α. A drop of extracellular pH from 7.4 to 6.0 for 5 s produced a rapid inward current in CHO cells transfected with ASIC3. The acidosis-induced currents were considered to be ASIC3 current, since they were blocked by APETx2 (2 μM), a specific ASIC3 blocker (Figure 3C). Unlike in rat DRG neurons, neither GW7647 (3×10^−8^M) nor PEA (10^−5^ M) had effect on I_pH6.0_ in ASIC3-transfected CHO cells (*P* > 0.1, post hoc Bonferroni's test, *n* = 10; Figure 3C and 3D). These results suggested that GW7467 and PEA failed to produce inhibitory actions on ASIC currents if there's no PPAR-α activity involved.

### Effect of GW7647 on proton- triggered action potentials of rat DRG neurons

**Figure 4:**
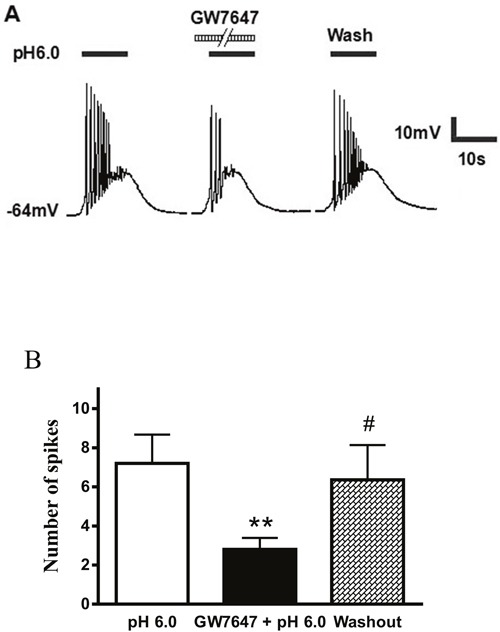
Effect of GW7647 on proton-evoked action potentials of rat DRG neurons **(A)**. Action potentials were evoked by a pH 6.0 acidic stimulus for 5 s with current-clamp recording in the presence of capsazepine (10 μM) or AMG 9810 (1 μM) to block proton-induced TRPV1 activation. The pretreatment of GW7647 (3×10^−8^ M, 2 min) decreased the acidosis-induced the number of action potentials. **(B)**. Bar graph shows the effect of GW7647 (3×10^−8^ M) on the number of action potentials produced by pH 6.0 acidosis perfusions. After 20 min washout of GW7647, acidosis-evoked action potentials recovered to control condition. ^**^P < 0.01, paired t-test, compared with pH 6.0 column alone; ^#^*P* < 0.05, paired t-test, compared with GW7647 + pH 6.0 column, *n* = 7 in each column.

ASIC activation triggers mainly sodium influx, depolarizes the membrane potential and excites neurons. We further investigated whether GW7647 had effect on proton-induced action potentials of rat DRG neurons. Under current-clamp conditions, a pH 6.0 stimulus for 5 s could trigger bursts of action potentials in a representative DRG neuron, even though TRPV1 was blocked by capsazepine (10 μM) or AMG 9810 (1 μM) (Figure 4A). Consistent with the results under voltage-clamp conditions, the acidosis-triggered action potentials were also suppressed by the pre-application of GW7647 (3×10^−8^M) for 2 min (Figure 4A). During exposure to pH 6.0 for 5 s, the mean number of action potentials was 7.20 ± 1.46 in seven DRG neurons tested. In contrast, pre-treatment of GW7647 (3×10^−8^ M) for 2 min decreased the mean number of action potentials to 2.82 ± 0.58 (*P* < 0.01, paired t-test; Figure 4B). After 20 min washout of GW7647, the mean number of action potentials triggered by acidic stimuli was 6.36 ± 1.78, which was not significant difference with control condition (7.20 ± 1.46, *P* > 0.1, paired t-test; Figure 4B). These results suggested that GW7647 reversibly inhibited bursts of action potentials induced by acidic stimuli in rat DRG neurons.

### Effects of GW7647 on nociceptive responses to injection of acetic acid and mechanical hypersensitivity induced by injection of CFA into the hind paw in rats

**Figure 5:**
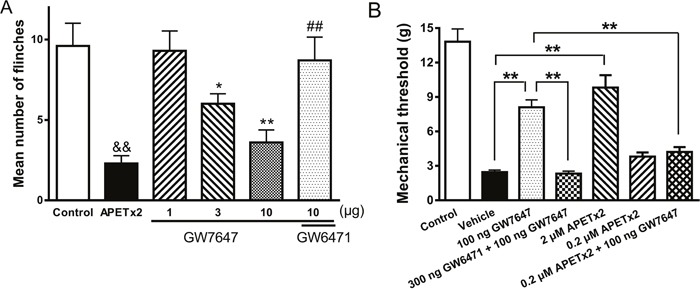
Effects of GW7647 on nociceptive responses to injection of acetic acid and mechanical hypersensitivity induced by injection of CFA into the hind paw in rats **(A)**. The bar graph shows that the nociceptive responses evoked by intraplantar injection of acetic acid (50 μl, pH 6.0) in the presence of the TRPV1 inhibitor capsazepine (100 μM). The acidosis-evoked nociception was blocked by pretreatment with APETx2 (50 μl, 20 μM), a blocker of ASIC3. The pretreatment of GW7647 decreased flinching behavior induced by acetic acid in dose-dependent manner (10-100 ng). The effect of GW7647 (100 ng) was blocked by co-treatment of GW6471 (300 ng). Each bar represents the number of flinches that animals spent licking/lifting the injected paw during first 5-min observation period (mean ± SEM of 10 rats in each group). ^&&^*P* < 0.01, ^*^P < 0.05, ^**^P < 0.01, Bonferroni's post hoc test, compared with control; ^##^*P* < 0.01, Bonferroni's post hoc test, compared with GW7647 (100 ng) column. **(B)**. Bar graph shows that effects of GW7647 and APETx2 on CFA-induced mechanical hypersensitivity in rats. Mechanical hypersensitivity was produced by injection of CFA into the left hind paw. Twenty-four hours following CFA injection, vehicle and drugs (50 μl) were pretreated into the inflamed hind paw. The pretreatment of GW7647 (100 ng) suppressed mechanical hypersensitivity. The effect of GW7647 (100 ng) was blocked by co-treatment of GW6471 (300 ng). Mechanical hypersensitivity was also suppressed by 2 μM APETx2, but not by 0.2 μM APETx2. The antihyperalgesic effect of GW7647 (100 ng) disappeared when co-treatment of 0.2 μM APETx2. Control column, baseline response before CFA injection. Mean ± SEM of 12 rats in each group. ^**^P < 0.01, Bonferroni's post hoc test.

Above results indicated that activity of ASICs was inhibited by GW7647 *in vitro*. We finally determined whether GW7647 inhibition of ASICs was involved in pain-related behaviors *in vivo*. Rats displayed an intense flinch/shaking responses after acetic acid (0.6%) was injected into hindpaws. The nociceptive responses mainly occurred during 0-5 min after intraplantar injection of acetic acid [16, 34]. We measured the number of flinches that rats spent licking and/or lifting the injected hindpaws. Figure 5A shows that nociceptive responses induced by acetic acid was potently blocked by pre-applied APETx2 (20 μM, 50 μl), a specific ASIC3 blocker, indicating the involvement of ASIC3 in the acidosis-induced nociception.

Pre-treatment of GW7647 dose-dependently alleviated the acidosis-evoked nociceptive responses (Figure 5A). Compared with control rats injected with acetic acid alone, rats displayed a significantly decreased flinch responses when medium- and high-dose (30 and 100 ng) of GW7647 were pre-applied into hindpaws. (Bonferroni's post hoc test, *P* < 0.05 and *P* < 0.01, *n* = 10). In contrast, the low dose (10 ng) of GW7647 had no effect on flinch responses (Bonferroni's post hoc test, *P* > 0.1, *n* = 10). Moreover, the alleviating effect of 100 ng GW7647 on acidosis-evoked nociceptive responses was blocked by co-application of 300 ng GW6471 (Bonferroni's post hoc test, *P* < 0.01, compared with 100 ng GW7647 alone, *n* = 10) (Figure 5A). These results suggested that periphery PPAR-α activation by GW7647 can relieve acidosis-evoked nociceptive responses in rats.

To further examine the physiological relevance of ASIC inhibition by GW7467, we used the rat CFA-induced inflammation model. CFA-treated rats developed mechanical hypersensitivity in the ipsilateral hind paw 24 h following injection of CFA, and the paw withdrawal thresholds to mechanical stimulation decreased (Figure 5B). Figure 5B shows effects of GW7647 and APETx2 on CFA-induced mechanical hypersensitivity in rats. Injection of 100 ng GW7647 into the inflamed hind paw resulted in a significant suppression of mechanical hypersensitivity (Bonferroni's post hoc test, *P* < 0.01, *n* = 12). The antinociceptive effect of GW7647 was blocked by co-treatment of 300 ng GW6471 (Bonferroni's post hoc test, *P* < 0.01, *n* = 12). CFA-induced mechanical hypersensitivity was also suppressed by 2 μM APETx2 (Bonferroni's post hoc test, *P* < 0.01, *n* = 12), but not by 0.2 μM APETx2 (Bonferroni's post hoc test, *P* > 0.1, *n* = 12). However, sub-analgesic dose of APETx2 (0.2 μM) blocked the antinociceptive effect of 100 ng GW7647 when GW7647 was co-treated with APETx2 (Bonferroni's post hoc test, *P* < 0.01, *n* = 12), suggesting the participation of ASIC3 in the antinociceptive effect of GW7647 on CFA-induced inflammatory pain.

## DISCUSSION

Our electrophysiological and behavioral evidence demonstrated that PPAR-α activation can acutely inhibit the activity of ASICs in nociceptive DRG neurons. PPAR-α agonist GW7647 decreased the amplitude of proton-activated currents and acidosis-triggered action potentials in rat isolated DRG neurons. Peripheral administration of GW7647 relieved acidosis-evoked nociceptive responses and CFA-induced mechanical hypersensitivity in rats.

As early as 1980, it was known that extracellular protons produce currents in primary afferents [35]. We found that lowering extracellular pH from 7.4 to 6.0 produced a rapid inward current in individual DRG neurons even though TRPV1 channels was blocked by capsazepine or AMG 9810. Most of these acidosis currents consisted of two parts, a large transient component and then a small sustained component [33]. These acidosis currents could be completely blocked by APETx2, a specific ASIC3 homomeric and heteromeric channel blocker. They were considered be ASIC3-like currents and mediated by ASIC3-containing channels, although precise ASIC subunits remained to be further identified. So far, at least six different ASIC subunits have been found in mammals [14]. All ASIC subunits besides ASIC4 subunit are expressed in DRG neurons [15, 18]. Especially, ASIC3 subunit predominantly expressed in nociceptors and was considered be a critical pH sensor. In primary sensory neurons, ASIC3 subunit is detected in cell bodies, axons and terminals, where its activation contributes to pain signaling [15–17].

We found that GW7647 exerted a rapid inhibiting effect on peak ASIC currents in rat isolated DRG neurons. The inhibiting effect of GW7647 on ASIC currents was blocked by PPAR-α antagonist, indicating involvement of PPAR-α. It has been shown that DRGs contain both PPAR-α mRNA and PPAR-α immunoreactivity in both small and large neuron [2]. In twenty neurons that are insensitive to GW7647, PPAR-α may be not expressed in these neurons recorded by ASIC currents. GW7647 has been found to attenuate the depolarization-evoked [Ca^2+^]_i_ in small DRG neurons through PPAR-α [36]. GW7647 shifted the proton concentration–response curve downward and decreased the maximum response without changing the IC_50_ values, indicating that PPAR-α activation resulted in a decrease in the efficiency of ASICs and had no effect on the affinity of ASICs for protons. ASICs are mostly permeable to Na^+^, activation of these channels can thus depolarize membrane potentials to the threshold of excitability and result in bursts of action potentials [37]. Data from current clamp experiments revealed that acidosis-triggered action potentials were also inhibited by GW7647. This decreased neuronal excitability may correlate with results that ASIC currents were attenuated by GW7647 in voltage clamp experiments. In the behavior experiments, we found that intraplantar administration of GW7647 relieved the acidosis-evoked nociceptive responses in rats in a dose-dependent manner. The GW7647 exerted an analgesic effect on acidosis-evoked nociceptive responses through PPAR-α, since its effect was blocked by PPAR-α antagonist. Obviously, the behavioral data corroborated the electrophysiological data and vice versa. The combined data strongly demonstrated that PPAR-α activation indeed inhibited the activity of ASICs, not only at the cellular level but also at the behavioral level. Pharmacological blockade of N-acylethanolamine acid amidase activity and preservation of endogenous PEA suppress visceral pain response elicited by intraperitoneal injections of acetic acid in mice through PPAR-α [38]. However, this visceral pain response is enhanced in female mice lacking PPAR-α [9].

PPAR-α, belonging to a nuclear receptor super-family, can function directly as a transcription factor that control gene transcription. Genomic effects of PPAR-α have latencies of at least 30 minutes (and up to days) and are associated with changes in protein synthesis [12]. Moreover, PPAR-α can also act at non-transcriptional targets to produce more rapid effects. PPAR-α may be involved in inhibition of ASIC activity by GW7647 and PEA, since their effects were blocked by PPAR-α antagonist and failed to emerge in CHO cells expressing ASIC3 but not PPAR-α. The current study revealed that activation of PPAR-α caused a rapid inhibition of ASICs within minutes in DRG neurons and intact animals. Effects of PPAR-α are too rapid to occur through classic transcription-dependent mechanisms, ASICs were therefore a non-transcriptional target of PPAR-α. Our previous study shows that activation of estrogen receptor-α, a member of the nuclear receptor super-family, can also exert a rapid potentiating effect on ASICs-mediated events through a non-genomic mechanism [39]. Studies have identified a number of rapid non-genomic effects of PPAR-α. For example, PPAR-α agonists rapidly engage peripheral vagal sensory fibers to reduce food intake in the small intestine, rapidly induce lipolysis and fatty-acid oxidation in liver and white adipose tissue [40, 41]. PPAR-α agonists exert also a rapid analgesia in a number of animal pain models through activating PPAR-α [2, 7]. Administration of PEA results in a rapid decrease in the elecrophysiological response of spinal nociceptors to peripheral formalin injection [2]. PPAR-α mediates acute effects of PEA on depolarization-evoked [Ca^2+^]_i_ in DRG neurons [36]. PEA-induced TRPV1 desensitization may be a potential molecular mechanism underling the rapid analgesic actions [42]. It has been shown that PPAR-α agonists regulate [Ca^2+^]_i_ signals via a rapid, non-genomic mechanism in sensory neurons and pancreatic β-cells [42, 43]. A significant fraction of the PPAR-α protein is even found to locate at the plasma membrane [43]. Alteration of [Ca^2+^]_i_ signals can cause a series of intracellular events. Whether these intracellular events are involve in the rapid inhibition of ASICs by activation of PPAR-α remained to be investigated. Although PPAR-α has not the evidence that PPAR-α is found on nerve endings, it is located in the cell bodies of DRG neurons. In current study, the cell bodies of DRG neurons were used as a simple and accessible model to examine the characteristics of peripheral terminals. Peripheral activation of PPAR-α by PEA rapidly suppresses input into wide dynamic range sensory neurons through modulating calcium-activated potassium channels [2].

The PPAR-α inhibition of ASICs may have a physiological significance in pathological conditions. During tissue injury and inflammation, protons are released from damaged cells and the de-granulation of mast cells, and extracellular pH value of the local area can lower to 5.4 [44, 45]. These released protons are sufficient to activate ASICs, subsequently trigger action potentials and produce pain signaling in primary afferents [45]. It has been shown that DRG neurons produce endogenous ligands for PPAR-α, such as PEA and OEA, which are sufficient to engage a substantial fraction of local PPAR-α [1]. When both ASICs and PPAR-α are co-expressed in peripheral sensory terminals, the current study indicated that endogenous ligands for PPAR-α could rapidly inhibited ASICs through activating PPAR-α, which further relieved acidosis-evoked nociceptive responses. Indeed, either activation of PPAR-α by GW7647 or blockade of ASIC3 by APETx2 suppressed CFA-induced mechanical hypersensitivity. Moreover, the antihyperalgesic effect of GW7647 disappeared when ASIC3 was blocked by APETx2, suggesting that ASIC3 mediated the antinociceptive effects of GW7467 on CFA-induced inflammatory pain.

Our results indicated that activation of peripheral PPAR-α rapid inhibited functional activity of ASICs in a non-genomic manner, which revealed a novel mechanism underlying rapid analgesia through peripheral PPAR-α. Targeting PPAR-α signaling may thus present new opportunities for the treatment of acidosis-mediated pain.

## MATERIALS AND METHODS

### Isolation of DRG neurons

The experimental protocol was approved by the animal research ethics committee of Hubei University of Science and Technology (No. 2016-68). All procedures accorded with international guidelines on the ethical use of animals, and we do our best to minimize the number of animals and their sufferings. DRG neuron preparation was performed as previously described [46, 47]. Briefly, 5-6 weeks old Sprague-Dawley male rats were decapitated following anaesthesia with ethyl ether. The rat DRGs were take out and transferred promptly into ice-cold Dulbecco's modified Eagle's medium (DMEM, Sigma). The connective tissues around DRGs were removed, and the DRGs were minced with fine spring scissors. The fragments of ganglions were transferred to a flask and incubated in 5ml of DMEM containing 0.5 mg/ml of trypsin (type II-S, Sigma), 1.0 mg/ml of collagenase (type I-A, Sigma) and 0.1 mg/ml of DNase (type IV, Sigma) for 25–30 min at 35°C in a shaking water bath. We finally added 1.25 mg/ml of soybean trypsin inhibitor (type II-S, Sigma) to suspend the trypsin digestion. Acute DRG neuron preparation was placed into a 35-mm Petri dish and kept for at least another 1 hour before electrophysiological recordings. The neurons used for electrophysiological recordings were 15–35 μm in diameter, which are thought to be nociceptive neurons.

### Cell culture and transfection

Cell culture and transfection was performed as previously described [48]. Briefly, ASIC3 cDNA was used for homologous expression in Chinese hamster ovary (CHO) cells. CHO cells were cultured at 37°C in a humidified atmosphere of 5% CO_2_ and 95% O_2_ and passaged twice a week. Transient transfection of CHO cells was performed using HilyMax liposome transfection reagent (Dojindo Laboratories). CHO cells were maintained in F-12 Nutrient Mixture (added 1.176 g of NaHCO_3_/L medium) supplemented with 10% fetal bovine serum and 1% gluta-MAXTM-1 (100×; Invitrogen). The ASIC3 cDNA was co-transfected at a 10 : 1 ratio with green fluorescent protein cDNA for identification of transfected cells at 4 μg per 35 mm dish with the use of Lipofectamine 2000. Electrophysiological recordings were performed 24–48 h after transfection.

### Electrophysiological recordings

Whole-cell patch clamp and voltage-clamp recordings were carried out with a MultiClamp-700B amplifier at room temperature (22–25°C) and analyzed using a Digidata-1440A A/D converter (Axon Instruments, CA, USA). DRG neurons were bathed in an external solution. External solution contained (in mM): 150 NaCl, 5 KCl, 2.5 CaCl_2_, 2 MgCl_2_, 10 HEPES, and 10 d-glucose, adjusted to 330 mOsm/L with sucrose and pH 7.4 with NaOH. Micropipettes were pulled using a Sutter P-97 puller (Sutter Instruments, CA, USA). The recording micropipettes were filled with internal solution. Internal solution contained (in mM): 140 KCl, 2.5 MgCl_2_, 10 HEPES, 11 EGTA, and 5 ATP, adjusted to 310 mOsm/L with sucrose and pH 7.2 with KOH. The resistance of the recording micropipette was in the range of 3–6 MΩ. To establish a whole-cell configuration, a positive pressure was applied through recording micropipette to form a gigaseal, and then a negative pressure was exerted to rupture membrane underneath the recording pipette tip. Capacitance and series resistance were compensated before recording the membrane currents. In voltage-clamp experiments, membrane voltage was maintained at −60 mV. In current-clamp recordings, only DRG neurons with a stable resting membrane potential (more negative than -50 mV) were used in this study. Signals were sampled at 10 to 50 kHz and filtered at 2 to 10 kHz, and the data were analysis using the pCLAMP 10 acquisition software (Axon Instruments, CA, USA).

### Drug application

All drugs were purchased from Sigma and used in the experiments include: hydrochloric acid, GW7647, GW6471, palmitoylethanolamide (PEA), complete Freund's adjuvant (CFA), capsazepine and or AMG 9810. All drugs were dissolved with the external solution before use and then reserved in a series of independent reservoirs connected to a linear array of silica tubes (o.d. / i.d. = 500 μm / 200 μm). The tube tips of drug application were positioned about 30 μm away from the recorded DRG cells. After opening the valve of the tube connection, the drug was applied by gravity. A rapid solution exchange (about 10 μl) could be achieved by shifting horizontally the tubes within about 100 ms. To functionally characterize ASIC activity, we added capsazepine (10 μM) or AMG 9810 (1 μM) to external solution to block TRPV1 activation in this study [29, 30].

### Nociceptive behaviour induced by acetic acid in rats

Rats were allowed to habituate in a 30 × 30 × 30 cm Plexiglas chamber for at least 30 min prior to nociceptive behavior experiments. Rats were firstly pretreated with vehicle, different dosages of GW7647, or GW6471 together with 50 μl capsazepine (100 μM) in right hind paw in separate groups. After 5 min, the dorsal face of the ipsilateral hind paw was subcutaneously injected acetic acid solution (0.6%, 50 μl) by the other experimenters using a 30 gauge needle connected to a 100 μl Hamilton syringe. After the acetic acid injection, nociceptive behavior (that is, number of flinches) was detected immediately over a 5 min period [16, 34].

### CFA-induced inflammatory pain model

Following determination of baseline thresholds, CFA (0.5 mg/ml saline) was injected into the left hindpaw of rats to induce peripheral inflammatory pain. Mechanical hypersensitivity was measured using von Frey filaments in 24 hours after CFA injection and 30 min after drug or vehicle application. When four of eight von Frey applications to the plantar surface of the hindpaw cause flexion withdrawal reflexes, the required minimum force was defined as the mechanical threshold.

### Data analysis

Data were expressed as mean ± SEM and statistically analyzed using the Student's t-test or analysis of variance (ANOVA), followed by Bonferroni's post hoc test. Concentration–response data were statistically analyzed using nonlinear curve-fitting program ALLFIT.

## Abbreviations

ANOVA: analysis of variance
ASICs: acid-sensing ion channels
CFA: complete Freund's adjuvant
CHO: Chinese hamster ovary
DRG: dorsal root ganglia
IC_50_: half maximal inhibitory concentration
I_pH_: proton-gated current
PEA: palmitoylethanolamide
pH_0.5_: pH for half-maximal activation
PPAR-α: peroxisome proliferator-activated receptor-α
OEA: oleoylethanolamide.
